# Death of Professor Joseph A. Eve

**Published:** 1886-02

**Authors:** 


### DEATH OF PROFESSOR JOSEPH A. EVE.

The demise of this eminent physician and philanthropist demands a more extended notice than our space at this time permits. The Medical Association of Georgia will doubtless soon pay fitting tribute to the memory of one of the most distinguished and best beloved of its former presiding officers. In advance of a more extended biographical sketch, which it is hoped will speedily appear, it is proper to remind the profession that for more than fifty years Dr. Eve has filled a most prominent place in their ranks.

In 1828, when he began his career in Augusta, Georgia, there was in the United States but one medical college south of Philadelphia. Thoroughly trained physicians and surgeons were comparatively scarce, and the obstacles and expense of obtaining a medical education so great that young men of suitable qualifications were deterred from engaging in that pursuit. Recognizing the public need of such an institution, the physicians of Augusta, with competent legal authority and liberal legislative pecuniary aid, founded the Medical College of Georgia.

Dr. Eve was chosen as one of the faculty upon its organization, and his name held a place upon the list for more than half a century. Of his early colleagues, several of whom are renowned in professional annals, all passed over the river before him, and left him a solitary, but most distinguished and venerated object of contemplation.

To his skill and fidelity as a teacher of the departments of the pan-science of medicine assigned him, thousands of his pupils, scattered all over the South and West, bear willing testimony. Two generations of the population of Augusta attest with universal acclaim his patience, integrity, tact and success as a general practitioner. All men and women, of all pursuits and conditions of society, award to him the crown as a hero of benevolence. If any modern man could, Dr. Eve might contest with St. Luke the proud title of nobility, "The Beloved Physician."

During the last few months of his life, Dr. Eve was engaged in the preparation of a series of articles embodying the result of his long study and observation in the departments of obstetrics and gynecology. This paper, the first of a series, has been for some time in our hands for publication, and the notes of others are now ready and have fortunately received the proper correction and approval.

Dr. Allen, who was for many years associated with Dr. Eve in practice, and who is therefore familiar with his views and methods, intends to carry out the original design, and to do this will draw largely from lectures in his possession, in which, Dr. Eve's experience on the subjects treated of, is fully recorded.

While these papers may be of some value to the profession generally, they will without doubt be of special interest to Southern practitioners, many of whom Dr. Eve was proud to rank among his pupils and to whom this will be his last and final teaching.

